# Lithium-ion battery aging dataset based on electric vehicle real-driving profiles

**DOI:** 10.1016/j.dib.2022.107995

**Published:** 2022-02-25

**Authors:** Gabriele Pozzato, Anirudh Allam, Simona Onori

**Affiliations:** Energy Resources Engineering, Stanford University, Stanford, CA 94305, USA

**Keywords:** Lithium-ion battery, EV driving-based data, Battery aging, Reference performance tests, Aging campaign, NMC 2170

## Abstract

This paper describes the experimental dataset of lithium-ion battery cells subjected to a typical electric vehicle discharge profile and periodically characterized through diagnostic tests. Data were collected at the Stanford Energy Control Laboratory, at Stanford University. The INR21700-M50T battery cells with graphite/silicon anode and Nickel-Manganese-Cobalt cathode were tested over a period of 23 months according to the Urban Dynamometer Driving Schedule (UDDS) discharge driving profile and the Constant Current (CC)-Constant Voltage (CV) charging protocol designed at different charging rates – ranging from C/4 to 3C. Ten (10) cells are tested in a temperature-controlled environment (23${}^{\circ }$C). A periodic assessment of battery degradation during life testing is accomplished via Reference Performance Tests (RPTs) comprising of capacity, Hybrid Pulse Power Characterization (HPPC), and Electrochemical Impedance Spectroscopy (EIS) tests. The dataset allows for the characterization of battery aging under real-driving scenarios, enabling the development of models and management strategies in electric vehicle applications.

**Specifications Table**

**Table:** 

Subject	Electrical and Electronic Engineering.
Specific subject area	EV Real-driving and diagnostic tests of lithium-ion batteries.
Type of data	Table.
How data were acquired	Hardware:
	• Arbin Instruments LBT21024 and Arbin measurement system;
	• Amerex IC500R thermal chamber;
	• Gamry EIS 1010E;
	• T-type thermocouple sensor, Omega.
	Software:
	• MITS Pro software and Data Watcher.
Data format	Raw and processed data.
Description of data collection	The Arbin system supplies the user-defined current profile to the battery cell and records the output voltage. A cycle is defined by the following Steps 1 to 6:
	1) CC charge at a constant C-rate of C/4, C/2, 1C and 3C until 4V;
	2) CV charge until current reaches the cutoff value of 50 mA;
	3) charge at C/4 until the cutoff voltage of 4.2V is reached (corresponding to 100% SOC);
	4) CV charge until current reaches the cutoff value of 50 mA followed by 30 minute rest;
	5) CC discharge at C/4 to bring the battery at 80% SOC;
	6) UDDS discharge to 20% SOC. Steps 1. to 6. are repeated.
	After either 25 or 50 cycles (consisting in Step 1. to 6.), RPTs, i.e., capacity test, EIS, and HPPC, are performed. The capacity test is performed at C/20 from a fully charged (i.e., 100%SOC) battery. To monitor the battery impedance as a function of the SOC and throughout the aging, EIS is performed at 20, 50, and 80% SOC. The temperature of the cells is regulated to 23 ${}^{\circ }C$ via the Amerex IC500R thermal chamber. In both raw and processed data, negative current defines discharge and positive current defines charge.
Data source location	Institution: Stanford Energy Control Lab, Energy Resources Engineering Department, Stanford University.
	City, State: Stanford, California.
	Country: United States of America.
	Latitude and longitude for collected samples/data:
	(37.426666918636386, -122.17397631867011).
Data accessibility	Repository name: Dataset_SECL_INR21700-M50T
	Data identification number (permanent identifier, i.e. DOI number): osf.io/qsabn
	Direct link to the dataset: https://osf.io/qsabn/?view_only=2a03b6c78ef14922a3e244f3d549de78

## Value of the Data

• The experimental campaign collects real-driving and diagnostic tests for ten INR21700-M50T NMC battery cells tested at 23${}^{\circ }$C and charged according to Constant Current (CC)-Constant Voltage (CV) charging protocol with CC charging rates of C/4, C/2, 1C and 3C.
• The discharging aging experiments are designed to mimic a typical electric vehicle real-driving pattern in the form of Urban Dynamometer Driving Schedule (UDDS) that brings the battery State of Charge (SOC) from 80% to 20%.
• Reference Performance Tests (RPTs), in the form of capacity test, Hybrid Pulse Power Characterization (HPPC), and Electrochemical Impedance Spectroscopy (EIS), are performed periodically to evaluate the cell degradation.
• The dataset provides EV real-driving aging cycling data that can enable robust development and fine-tuning of battery aging models for health estimation strategy design and model-based diagnostic methods.
• To the best of the authors’ knowledge, this dataset is the first of its kind as it provides battery aging data from EV real-driving scenarios.

## 1 Data Description

The dataset is composed of EV real-driving profiles and RPTs for ten INR21700-M50T NMC cells over a period of 23 months. Technical specifications of the cells are summarized in Table 1.

**Table 1:** Technical specifications INR21700-M50T NMC cell [2].

Manufacturer	LG Chem	
Model	INR21700-M50T	
Positive electrode	LiNiMnCoO2	
Negative electrode	graphite and silicon [1]	
Size (diameter×length)	21.44 mm×70.80 mm	
Weight	69.25g	
Nominal capacity ($Q_{nom}$)	4.85Ah	
Nominal voltage	3.63V	
Charge cutoff voltage	4.2V	
Discharge cutoff voltage	2.5V	
Cutoff current	50mA	

**Fig. 1:**
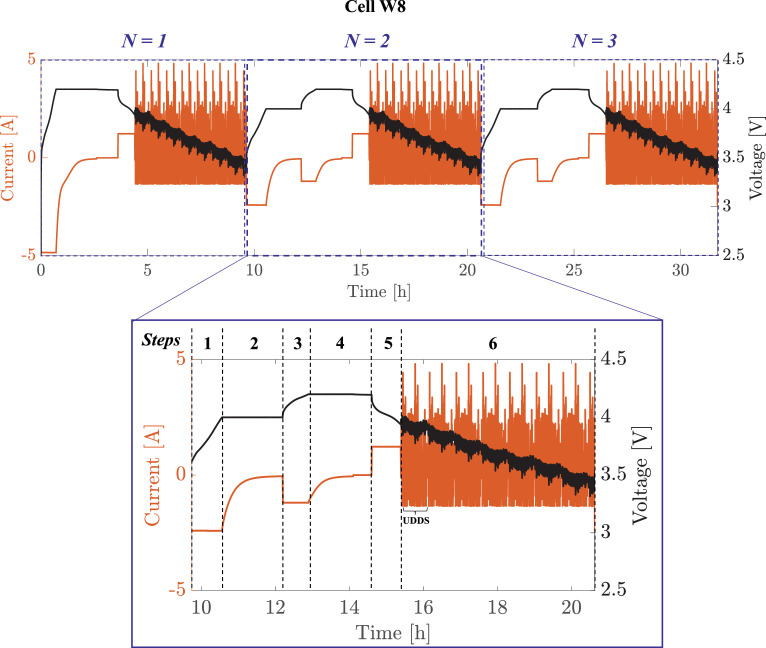
First three cycling profiles for cell W8 after the first RPT. According to Table 3, charging is performed at C/2. The charging profile for N=1 is such that the battery is charged at 1C CC until voltage reaches 4.2V, then one hour of rest time follows the CV charging. In the cycles N=2 and N=3, instead, the charging profiles follow the protocol outlined in Step 1 through 4 of Table 2. In the zoomed window, the 6 steps for the *Cycle* are streamlined, as defined in Table 2. Positive and negative currents are for discharge and charge, respectively.

To reproduce the aging experienced by the lithium-ion cells during real-world EV operation, the charging/discharging profiles shown in Fig. 1 were used. A *Cycle* is composed by the sequence of 6 steps, listed in Table 2. A *Cycle* starts with a CC charge performed at a C-rate of C/4, C/2, 1C, or 3C, as specified in the second column of Table 3 (Step 1). Once the battery voltage reaches 4V, a CV phase starts (Step 2) until the current goes below 50mA. Next, Step 3 (CC at C/4) and Step 4 (CV) are designed to bring the battery to 4.2 V, corresponding to 100% SOC. Step 5 is used to discharge the battery from 100% to 80% SOC at C/4 constant current. In Step 6, a concatenation of UDDS cycles is used to discharge the battery from 80% to 20%. The driving profile is the same used in Fig. 6 of Allam and Onori [3] normalized to the cell capacity used in this work. After each RPTs, the cells are brought to 100% SOC via 1C CC charge followed by CV until the current is below 50mA and left at rest for one hour (see, Fig. 1 the plot for N=1).

**Table 2:** Description of the experimental *Cycle*.

Step	Action	Exit condition
1	CC charge at C-rate specified in the second column of Table 3	4V is reached
2	CV charge	Current below 50mA
3	CC charge at C/4	4.2V is reached
4	CV charge followed by 30 minute rest	Current below 50mA
5	CC discharge at C/4	20% discharged capacity (80% SOC)
6	UDDS discharge	60% discharged capacity (20% SOC)

**Table 3:** Cells label, test charging condition, temperature and diagnostic test number. For each diagnostic test, the number of cycles experienced by the cell is reported. All cells are tested at 23${}^{\circ }C$.

			Diagnostic test [cycle]								
Label	Charge C-rate	Environment temperature [${}^{\circ }$C]	#1	#2	#3	#4	#5	#6	#7	#8	#9
W3	3C	23	0	25	75${}^{*}$	-	-	-	-	-	-
W4	C/4	23	0	25	75	123	132	159	176	179	-
W5	C/2	23	0	25	75	125	159	167	187	194	219
W7	C/4	23	0	25	75	125	141${}^{*}$	-	-	-	-
W8	C/2	23	0	25	75	125	148	150	151	157	185
W9	1C	23	0	25	75	122	144	145	146	150	179
W10	3C	23	0	25	75	122	146	148	151	159	188
G1	3C	23	0	25	30	37	62	-	-	-	-
V4	C/4	23	0	20	45	70	95	-	-	-	-
V5	1C	23	0	12	18	29	-	-	-	-	-

* the cell was dismissed and the aging campaign terminated.

The diagnostic tests, i.e., capacity, EIS, and HPPC tests, are run periodically (for the majority of the cells every 25 cycles, see Table 3). Capacity test, performed at C/20 discharge from a fully charge cell, is used to evaluate the cell discharged capacity, HPPC is used to evaluate the battery high frequency resistance at different SOC, and EIS is performed to assess the battery impedance between 0.01Hz and 10kHz at 20%, 50%, and 80% SOC.

**Fig. 2:**
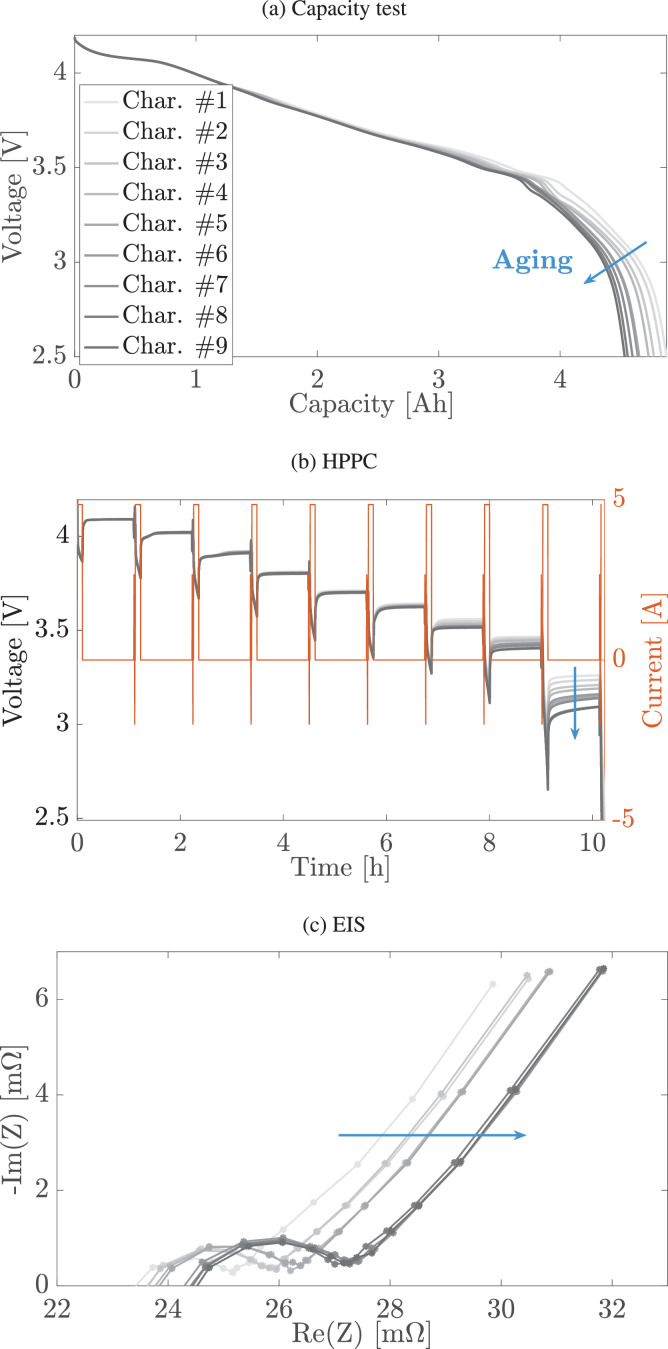
RPTs for cell W8: (a) capacity test at C/20 CC, (b) HPPC, and (c) EIS at 50% SOC. Light-blue arrows indicate where the signals move upon aging. In (b), positive current indicates discharge and negative current charge, respectively.

Aging leads to a reduced discharged capacity and increased impedance, as shown from capacity tests in Fig. 2a and EIS tests Fig. 2c, respectively. At the same time, from the HPPC tests in Fig. 2b one can observe an accentuated voltage drop due to increased impedance at low SOC as the aging progresses. Plots of Fig. 2 are for cell W8.

Table 3 reports on the total RPTs performed on the tested cells until February 1st, 2022 at various Crate during charging. Between one diagnostic test and the next, cells are cycled according to the procedure described in Fig. 1. For each RPT, the number of cycles reached by the cell is reported. The first RPT (#1) is performed before starting the aging cycling campaign and provides information on the pristine cells. For cells W5, W8, W9, and W10 9 diagnostic tests were performed. Cell W4, G1, V4, and V5 have a lower number of RPTs because the aging campaign was started later. A few off-trend situations have been recorded. The calculated impedance of W3 from the HPPC test was approximately twice as high as the impedance of the other cells, which led to the aging campaign for this cell to be terminated. In the case of cell W7, tests were stopped because impedance measurements exhibited inconsistencies, wherein a lack of any physically meaningful trend was observed as the cell aged.

**Fig. 3:**
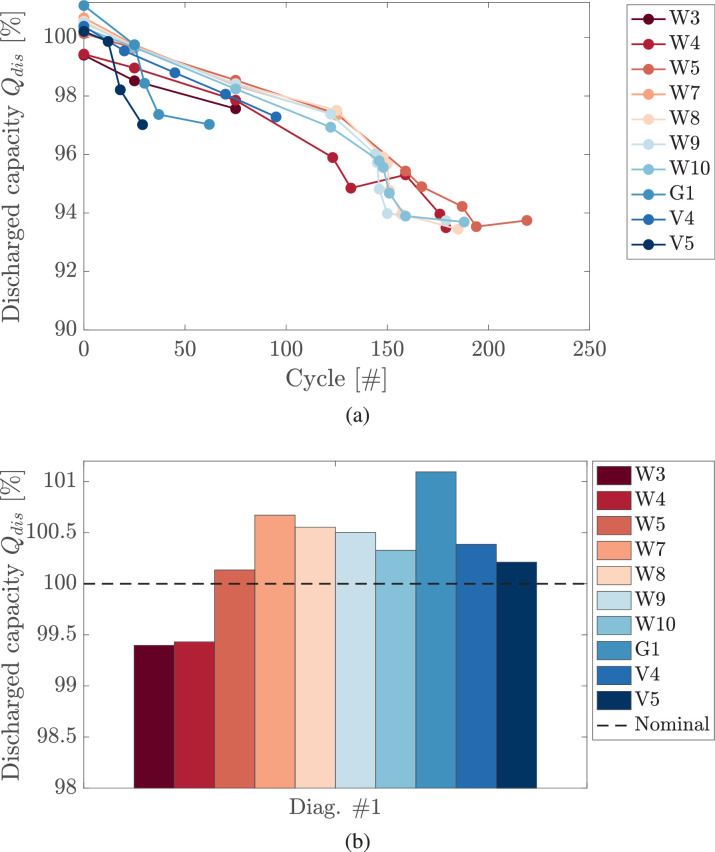
(a) capacity fade curves for the tested cells. Discharged capacities are computed from capacity tests according to Eq. (1). In the bottom, the distribution of the discharged capacity for pristine cells (diagnostic test #1).

For each cell, discharged capacities are calculated from the capacity tests performed at each RPT. The discharged capacity, measured in Ah, and normalized with respect to $Q_{nom}$ (defined as in Table 1), is computed integrating the current I(t) with respect to time:

(1) $$Q_{dis}=\frac{\frac{1}{3600}\int I\left(t\right)\;dt}{Q_{nom}}\times 100\;\left[\%\right]$$

with 3600 the seconds to hours conversion factor. Capacity tests are performed at C/20 CC with I(t) constant and equal to 0.24A. Discharged capacity curves for each cell are shown in Fig. 3(a).

### 1.1 Dataset structure

The dataset provides both raw (*.xlsx*) and processed (*.mat*) data. Raw data are saved in excel spreadsheets, that can be be used to extract raw diagnostic and cycling data. The main limitation of using the raw data is the large size (248.9 GB for the whole dataset), that prevents fast data analysis and processing. To allow for fast data analysis, relevant signals are extracted from raw data and saved in *.mat* files, this operation reduces the size of the overall dataset down to 93.7%. It is worth mentioning that data inside *.mat* files are neither filtered nor resampled.

**Fig. 4:**
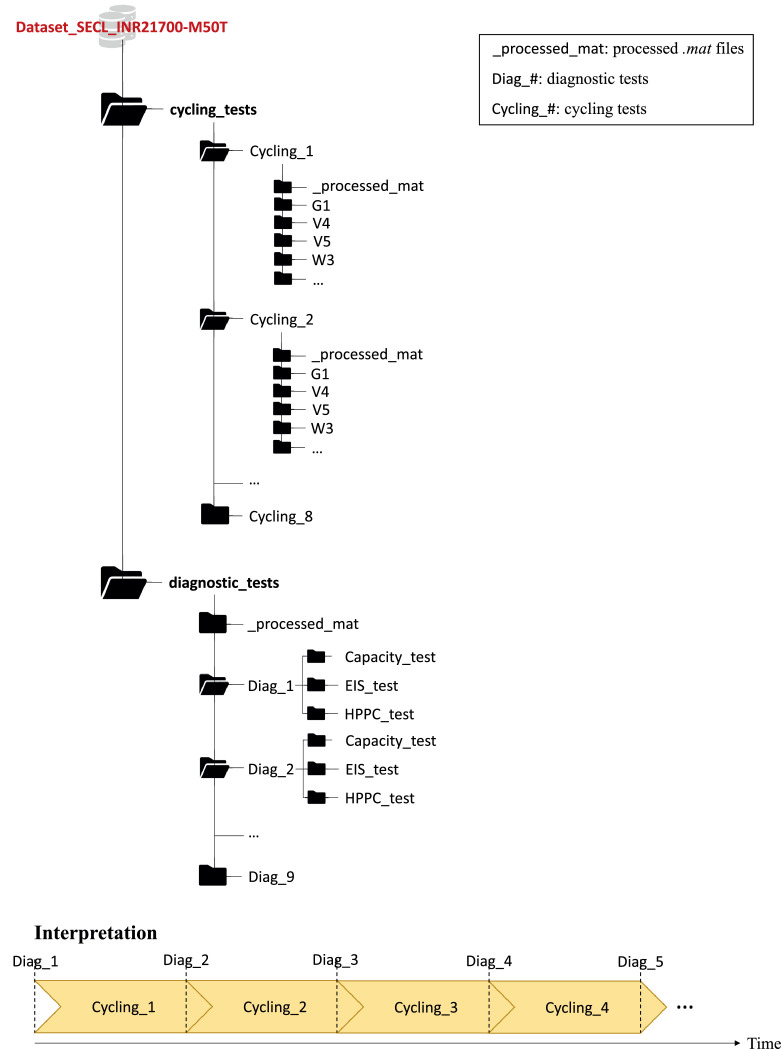
Dataset folder structure.

The dataset folder, available online (as specified in the “Data accessibility” field), is structured as in Fig. 4. The parent folder Dataset_ SECL_INR21700-M50T has two sub-directories: cycling_tests and diagnostic_tests.

The folder cycling_tests contains the aging cycling data for all the cells. Cycling data are divided into the folders Cycling_# (with # = 1,...,8). Each folder Cycling_# collects both raw data, divided by cell (i.e., G1, V4, etc.), and processed data, inside _processed_mat. Raw cycling tests are composed of several *.xlsx* files, that must be merged for the analysis. The *.mat* files are obtained after merging raw *.xlsx* files and are available to the user. Inside the folder _processed_mat, the Matlab script data_analysis.m is provided to plot voltage and current profiles.

As shown in Fig. 4 (bottom), between two cycling folders, RPTs are performed and collected into diagnostic_tests. Raw data for each RPT are divided into folders named Diag_# (with # = 1,...,9). For example, the diagnostic test #1 in Table 3 corresponds to Diag_1. Each folder Diag_# contains capacity, EIS, and HPPC tests inside the subfolders Capacity_test, EIS_test, and HPPC_test, respectively. The subfolder _processed_mat inside diagnostic _tests collects the processed *.mat* files and the Matlab file data_analysis.m for the analysis of voltage, current, and impedance.

## 2 Experiment Design, Materials and Methods

**Fig. 5:**
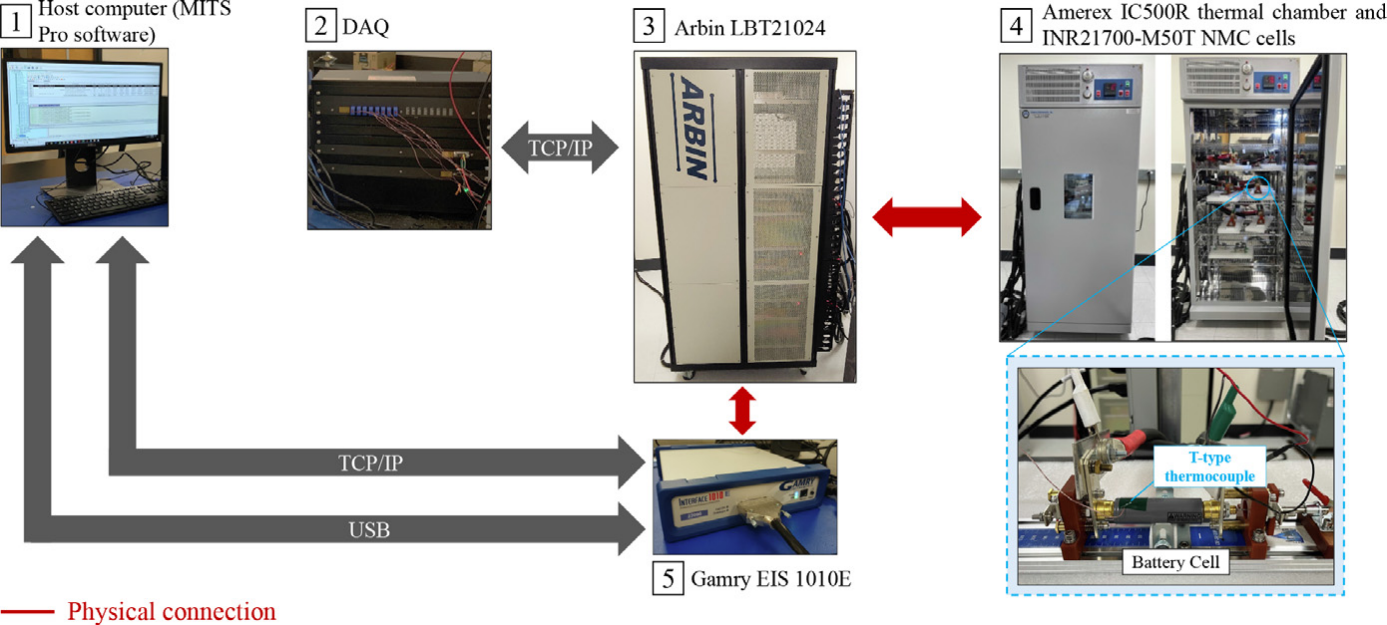
Equipment available at the Stanford Energy Control Lab (https://onorilab.stanford.edu/).

Cycling and diagnostic experiments are performed with the equipment available at the Stanford Energy Control Lab (Fig. 5). Both cycling and diagnostic tests are designed with the MITS Pro software , which allows to define protocols, i.e., the sequence of steps to be followed in order to perform an experiment. The Data Acquisition System (DAQ)  is interfaced with Arbin LBT21024 , which generates and inputs the desired current profile to the ten INR21700-M50T NMC cells tested and measures the output voltage. Each cell is tested inside the Amerex IC500R thermal chamber  and instrumented with a T-type thermocouple to measure the surface temperature in the center location. The Gamry EIS 1010E is connected to the Arbin LBT21024 and MITS Pro (via USB link) and used to perform EIS tests at different SOC, namely, 20, 50, and 80% .

Each test is exported in *.xlsx* files, raw data structures that can be conveniently converted into *.mat* files.

## Ethics Statement

Hereby, we Simona Onori, Anirudh Allam, and Gabriele Pozzato assure that for the manuscript *Lithium-ion battery aging dataset based on electric vehicle real-driving profiles* the following is fulfilled:

1. This material is the authors’ own original work, which has not been previously published elsewhere.
2. The paper is not currently being considered for publication elsewhere.
3. The paper reflects the authors’ own research and analysis in a truthful and complete manner.
4. The results are appropriately placed in the context of prior and existing research.
5. All sources used are properly disclosed. Literally copying of text must be indicated as such by using quotation marks and giving proper reference.
6. All authors have been personally and actively involved in substantial work leading to the paper and will take public responsibility for its content.

## CRediT authorship contribution statement

**Gabriele Pozzato:** Formal analysis, Data curation, Writing – original draft. **Anirudh Allam:** Methodology. **Simona Onori:** .

## Declaration of Competing Interest

The authors declare that they have no known competing financial interests or personal relationships that could have appeared to influence the work reported in this paper.

## References

[1] Steinhardt M., Gillich E.I., Rheinfeld A., Kraft L., Spielbauer M., Bohlen O., Jossen A. (2021). Low-effort determination of heat capacity and thermal conductivity for cylindrical 18650 and 21700 lithium-ion cells. J. Energy Storage.

[2] LG Chem, Product specification, rechargeable lithium ion battery, model: INR21700 M50T 18.20 Wh, 2018, (https://www.batteryspace.com/prod-specs/11514.pdf).

[3] Allam A., Onori S. (2021). Online capacity estimation for lithium-ion battery cells via an electrochemical model-based adaptive interconnected observer. IEEE Trans. Control Syst. Technol..

[4] Catenaro E., Onori S. (2021). Experimental data of lithium-ion batteries under galvanostatic discharge tests at different rates and temperatures of operation. Data Brief.

